# Case Reports: Multifaceted Experiences Treating Youth with Severe Obesity

**DOI:** 10.3390/ijerph16060927

**Published:** 2019-03-14

**Authors:** Karen E. Schaller, Linda J. Stephenson-Somers, Adolfo J. Ariza, Maheen Quadri, Helen J. Binns

**Affiliations:** 1 Pediatrics, Ann & Robert H. Lurie Children’s Hospital of Chicago, Chicago, IL 60611, USA; AAriza@luriechildrens.org (A.J.A.); MQuadri@luriechildrens.org (M.Q.); HBinns@luriechildrens.org (H.J.B.); 2 Center on Obesity Management and Prevention, Mary Ann & J. Milburn Smith Child Health Research, Outreach, and Advocacy Center, Stanley Manne Children’s Research Institute, Chicago, IL 60611, USA; LSomers@luriechildrens.org; 3 Clinical Nutrition, Ann & Robert H. Lurie Children’s Hospital of Chicago, Chicago, IL 60611, USA; 4 Pediatrics, Feinberg School of Medicine, Northwestern University, Chicago, IL 60611, USA

**Keywords:** pediatrics, severe obesity, child protective services, psychosocial

## Abstract

The management of youth with severe obesity is strongly impacted by social determinants of health and family dynamics. We present case studies of three patients seen in our tertiary care obesity treatment clinic as examples of the challenges faced by these patients and their families, as well as by the medical team. We discuss how these cases illustrate potential barriers to care, the role of child protective services, and we reflect upon lessons learned through the care of these patients. These cases highlight the need for comprehensive care in the management of youth with severe obesity, which can include: visits to multiple medical specialists, and mental and behavioral health providers; school accommodations; linkage to community resources; and, potentially, child protective services involvement. Through the care of these youth, our medical team gained more experience with using anti-obesity medications and meal replacements. The care of these youth also heightened our appreciation for the integral role of mental health services and community-based resources in the management of youth with severe obesity.

## 1. Introduction

Treatment of obese children involves family-wide change to increase physical activity and improve dietary habits; yet, such treatment has limited success [1,2]. There is an ever growing appreciation that social determinants of health (neighborhood and built environment, economic stability, education, social and community context, and health and healthcare) can greatly impact health [3,4,5], and can interfere with the improvement in health outcomes that are expected in response to the delivery of health care. For example, living in unsafe environments and lack of access to facilities may lower the ability of children to increase their activity levels [6]. Even when resources are available, individuals with low levels of education may be less likely to use a recommended strategy [7].

Factors such as living environment, family life experiences, levels of parental support, and peer relationships can impact a youth’s ability to make healthier dietary choices, reduce screen time and be physically active [7,8]. By modeling healthy dietary behaviors, having healthy foods in the home and having family meals, parents/caregivers can promote healthier choices for their children [6,8,9,10].

Patient excess adiposity causes structural and functional abnormalities [11] that can impair movement and lead to inability to perform routine activities of daily living. Excess adiposity can also impact brain response to visual and oral stimuli [12,13], making healthy dietary choices more difficult to achieve. In addition, patient mental health problems can be integrally related to excess weight gain, including issues such as disordered eating, eating in response to emotions or use of psychotropic medications; or mental health problems may develop as a consequence of obesity, sometimes related to bullying [14,15,16].

Genetic inheritance, prenatal exposures and early life nutrition can also impact a child’s weight trajectory and adult weight [17,18,19,20,21]. Prenatal exposures can cause intellectual and behavioral impairments that pose additional challenges when managing children with obesity [22,23].

Obesity treatment efforts focusing narrowly on behavior change of diet and physical activity patterns [24] may accomplish little, if the myriad problems of the child and family are not also identified and addressed. Oftentimes to address these many factors (e.g., neighborhood safety, housing, living environment, school, family cohesion, parental/caregiver health and behaviors, finances, and mental health issues) a comprehensive team is needed. Tertiary care pediatric obesity treatment providers have sometimes reached out to child protective services (CPS) to gain support for the health behavior change process [25]. CPS has the ability to identify and address issues that are beyond the reach of providers in the tertiary care setting. The aim of this paper is to present three case studies that convey the complexity of the circumstances of youth with severe obesity and the multifaceted aspects of their care. To highlight connections between the medical care systems and community resources, we describe youth who had CPS involvement during the course of care delivery.

## 2. Materials and Methods

At our pediatric obesity treatment clinic, we provide patient- and family-centric care with a team of medical providers, nutritionists, and social workers. Approximately two-thirds of the patients we see have severe obesity [24]. At visits, we assess patient diet and physical activity and identify and address family, community, and psychosocial issues that may be barriers to treatment. We use motivational interviewing techniques to help patients and families set goals to overcome barriers to increasing physical activity and making improved dietary choices. At follow-up visits, we review progress and identify and address barriers to change. Most patients have return visits every 3 months, but visit frequency may vary. We partner with specialists to assess and manage the serious medical and psychiatric conditions that often accompany excess adiposity. While our treatment has a modicum of success, on par with other tertiary care pediatric obesity programs [26], for our most complex patients we struggle to provide care that leads to improved weight status and to address the many issues identified. Clinical management challenges may be related to poverty, poor housing, learning difficulties, low education, transportation, unsafe neighborhoods, and school issues. We sometimes reach outside our clinical setting to obtain the resources needed to overcome these challenges.

The youth in this report were selected from among those we have seen with CPS involvement, because they portray the difficult challenges of care and need for care coordination. We selected cases with varying CPS management strategies and varying outcomes. The Lurie Children’s Hospital Institutional Review Board determined that this project (#2019-2623) does not meet the definition of human subject research. To preserve anonymity, information on weight status changes over time is conveyed by presenting percent of the 95th BMI percentile for age and sex (BMIp95) [24]. This is the preferred measure for tracking weight-related change in youth with obesity [27]. For Case 2, we also present percent body fat (%BF) measurements [28]. Percent body fat measurements were not available for the other youth.

## 3. Case 1

When she first presented to our clinic, AA, a teenage girl with severe obesity (BMIp95 302%), was living with her mother and siblings. The family had limited resources. She was attending school regularly. Her medical history included elevated liver enzymes, polycystic ovarian syndrome (PCOS), and obstructive sleep apnea. She had a sleep device system for at home use but was noncompliant, and she had already failed multiple appointments for an endocrinology evaluation. At the visit, she was noted to have hypertension and anxiety. We also identified interaction issues with her mother and other adults, including possible past physical abuse. Therapy and psychiatric services were recommended.

Over the next 9 months, she was seen monthly at our obesity treatment program. We initiated metformin to treat PCOS. During this time the state Medicaid structure changed to a managed care system and she lost access to her primary care provider. She continued to gain weight (BMIp95 347%). She was later diagnosed with depression and anxiety and stopped attending school. One year after starting services at our clinic she continued to gain weight rapidly (BMIp95 362%). She failed appointments for sleep apnea treatment and stopped taking prescribed medications. This prompted our first call to CPS. The CPS evaluation revealed a need for additional therapy for depression. The CPS case worker tried to arrange an admission to an outside psychiatric hospital, which was rejected due to AA’s weight and medical issues. We were able to obtain urgent outpatient psychiatric services (therapy and medications) through an emergency department evaluation. A few days later, obesity clinic providers arranged a medical admission for treatment of sleep apnea and hypertension. She was discharged home on medications and a new sleep device. At a clinic visit 3 weeks post-discharge, CPS was called again due to substantial weight gain. We tried to identify structured residential programs which could provide an environment conducive to weight loss. We also started biweekly visits. Additionally, CPS conducted biweekly home visits. AA responded well to close monitoring; her weight dropped, she was more active, using her sleep device and attending follow-up visits with specialists. She transitioned to a community-based facility close to her house for weekly psychiatric care. The CPS case worker identified additional resources to help the family with housing issues.

A few months later, the CPS case worker stopped communicating with our team. AA lost psychiatric services again due to facility closure and was re-connected to psychiatric care at our medical system. Her PCOS and pre-diabetes care transitioned to the endocrinology team. Due to metformin nonadherence, and a rising hemoglobin A1c level, she was hospitalized by endocrinology for diabetes education (this was 1.5 years after her first obesity treatment clinic visit; BMIp95 358%). Metformin was not restarted due to marked rise in her liver enzymes. Monthly visits were restarted in our obesity treatment program and she continued receiving psychiatric services. Despite being off metformin and never initiating home insulin use, her weight remained stable. Our social worker (SW) met with the family at every obesity treatment follow-up visit to identify and address needed resources. A liver biopsy led to diagnosis of autoimmune hepatitis.

AA met daily with her school-based counselor and established care at a community-based facility where she met with a therapist weekly and had care oversight provided by a psychiatrist. Following consultation with our team, the psychiatrist initiated additional medication to lower appetite (BMI95 353%).

There was increasing conflict between mother and AA and we considered options for alternative living environments. We investigated summer camps and boarding schools for teen patients with obesity; all facilities denied services due to her weight and health conditions. At about 2 years into our care, AA began meeting with the bariatric surgery team monthly, and she was seen every 2 weeks at our clinic. She continued frequent meetings with her counseling services. She was taking her medications and her weight remained stable.

Several months later, her weight was up again. The bariatric surgery team suggested initiating a liquid meal replacement plan which required weekly monitoring visits. AA began liquid meal replacement enthusiastically. Liquid meal replacement instructions were to mix 1 pouch in water and drink one in place of breakfast and one in place of lunch with a well-balanced, low calorie dinner. As treatment continued, mother expressed difficulty complying with the frequency of visits. The SW assisted with transportation, and we lessened visit frequency. Her school counselor and outside therapist both contacted us to relay AA’s concerns about the liquid meal replacement, her high levels of stress, and anxiety surrounding bariatric surgery. She was increasingly uncooperative and belligerent with medical providers. After a few weeks on the liquid meal replacement, she stopped use and stopped care with the bariatric surgery team, but continued visits to the obesity treatment clinic. She reverted back to unhealthy eating behaviors, including binge eating, excessive portions, and selection of calorie dense, low nutrient foods.

Conflict with her mother increased and AA started missing school. At this point, AA had essentially given up on making changes. In addition, AA stopped attending her counseling sessions and so was discharged from weekly counseling. At her last visit to our obesity treatment clinic (BMIp95 371%), she was upset and tearful. She did not want to know her weight. Her mother expressed that she did not have any control over her daughter. We reported to the family that we were planning to place another call to CPS (our third call in 2.5 years). Mother and AA did not return for any more visits to our program. We don’t know the outcome of our CPS call.

## 4. Case 2

BB was a preteen at his first obesity treatment visit (BMIp95 173%, 52.2% body fat). He was placed in foster care as an infant following a term birth to a cocaine-abusing mother. BB had cognitive impairment, severe behavioral challenges, and limited self-care skills. His difficult behavior was managed with an antipsychotic drug that can promote weight gain; his educational plan included a one-on-one aide when in school. He had attention deficit hyperactivity disorder, but stimulant medication had not been approved by his insurance. He was also receiving asthma maintenance therapy and had constipation with encopresis and nocturnal enuresis.

The foster parents had been concerned about his weight for the past 3 years and had started making changes just prior to the visit. They reported offering fruits for snacks, but he would choose the chips. They had started locking the refrigerator, as he was sneaking food and eating at night. He was reported to be a very picky eater, with a diet including almost no vegetables. They had also just started to address the constipation/encopresis management per primary care provider recommendations, and we referred him to gastroenterology for further management. Our first visit recommendations included household-wide changes to promote healthy foods for the entire family.

At a second visit, 2 months later, he had gained weight (BMI95p 175%, 53.5% body fat). BB was now taking a stimulant medication, but recommendations from the prior visit had not been implemented. His foster parents had health challenges of their own, which impacted their ability to make the suggested changes.

Upon return to our obesity treatment program, 5 months after his initial visit, BB had gained weight (BMI95p 178%, 57.5% body fat) and was noted to have bowed legs. He was receiving the same medications and was also taking an oral steroid burst for asthma control. The family reported that due to busy schedules they were cooking less at home. He still was not eating vegetables. BB was sent for radiographic evaluation: hip films were normal, but his right knee had findings consistent with Blount’s disease. The clinic provider called the primary care pediatrician and referred BB to a pediatric orthopedic surgeon.

At the next visit, 4 months later, we learned that for the past 2 months he had been living in a different foster home. Our team was not involved with this change. His weight improved (BMIp95 161%, 49.9% body fat). His knee was in a brace; responding well to treatment. The CPS case worker accompanied him to the visit and reported dietary improvements (eating more fruits and vegetables) along with continuing concern about his volatile moods. His new foster parents had implemented structured outdoor play and were providing healthy foods. His encopresis had stopped and he had started bathing himself.

At the next visit, 1.5 years after his first visit, he continued to do well (BMIp95 148%, 44% body fat). We learned that he had experienced a psychiatric crisis with erratic and aggressive mood swings, threatening harm to self and foster parents. He required hospitalization, was stabilized, and returned to the second foster family. His behavior was being managed with additional medications.

We next saw BB 10 months later, his weight status continued to improve (BMIp95 130%, 28.7% body fat). Due to additional psychiatric problems and threatening behaviors he had recently been moved into a group home setting. He reported reduced opportunities for activity, but was doing push-ups 5 days/week.

Our last visit with BB was 5 months later (BMIp95 129%, 28.6% body fat), almost 3 years since his initial visit. He was still living in the group home. He was usually getting just 1 fruit and 1 vegetable serving daily. He no longer needed a leg brace. He was physically active, primarily through school gym, but also reported doing exercises in his room. The CPS case worker who accompanied him to the visit was not familiar with his dietary or physical activity history.

## 5. Case 3

CC was born extremely premature; he required nasogastric tube feedings for his first 6 months due to aspiration. He first presented for primary care services to our medical system in his preschool years (BMIp95 170%). The primary care provider identified speech and developmental delays. He also had asthma and snoring. He was using a bottle and had advanced untreated caries. The nutritionist identified parental feeding strategies (i.e., bottle use, chocolate flavoring of milk) aimed at keeping CC from crying. Over the course of several primary care visits he continued to gain weight and was referred to speech, nutrition, dental, ophthalmology, and otolaryngology. A sleep study showed significant oxygen desaturations during sleep, necessitating direct admission to the intensive care unit (ICU). An urgent tonsillectomy and adenoidectomy were performed. He was followed for 5 more months (still gaining weight), then the family transferred care outside of our medical system.

Five years later, he was seen twice by a cardiologist at our medical system for a cardiac evaluation prior to starting an exercise program (BMIp95 274%); he was cleared for exercise. His next contact with our institution was 2 years later as a young teen (BMIp95 301%) when he was admitted to our ICU for an asthma exacerbation; he was not using his prescribed home sleep device. During the admission, he had hypertension and was diagnosed with diabetes. He received diabetes education, was started on medications, including metformin, and reinitiated use of his sleep device. The ICU team called CPS to initiate home monitoring and compliance with specialist care.

He was seen for a first visit at our obesity treatment clinic 3 months after hospital discharge (BMIp95 300%) and reportedly he was using his home sleep device and taking his medicines, but had not been attending school. He failed follow-up appointments with various specialties over the next 1.5 years. We are unsure of CPS involvement over this period.

Three years after the ICU admission, CC presented to an outside hospital emergency department for shortness of breath, abdominal pain, swelling of one leg with inability to ambulate. He was transferred to our ICU; his weight had increased substantially (BMIp95 415%). He had stopped using his sleep device for the prior 9 months. He was hospitalized for 5 weeks to treat a presumed lower extremity blood clot (imaging studies were inconclusive due to his body habitus), and managed for pulmonary hypertension and right ventricular heart failure. The father disclosed parental health concerns which may have limited parent’s ability to appropriately care for CC. The ICU team again called CPS to implement a plan to transition to a short term rehab facility and identify a foster home placement. Attempts were made to find placement for CC, but no facility would accept a patient with such severe medical conditions. The parents wanted CC to remain under their care. CC was discharged (BMIp95 306%) to parents’ care with close supervision by CPS. CPS arranged for transportation to his multiple outpatient visits.

He was seen shortly after hospital discharge in our obesity treatment clinic. He was receiving psychiatric services and had follow-up visits with various specialists. He attended school 1 day weekly and completed online schooling the other days. The CPS case was closed after 6 months of supervision. Transportation to appointments became a significant problem. He was seen for a third obesity treatment clinic visit 8 months post-hospital discharge. His weight had improved (BMIp95 268%), but he was using his sleep device intermittently. He was discharged from psychiatric care due to missed appointments, and didn’t show for visits to other specialists. The SW provided information on obtaining reduction in transit fares, but the family was unable to follow through with recommendations.

At a follow-up visit with cardiology (20 months post-hospitalization) his weight was up (BMIp95 277%); the cardiologist threatened calling CPS due to missed appointments, non-compliance with his home sleep device, and drinking 4–5 cups of juice per day. In his next visit to our clinic, one month later, CC’s weight had improved (BMIp95 271%). At 2 subsequent bi-monthly clinic visits we continued to reinforce goals, such as how to choose drinks that had no sugar. We re-referred him to sleep medicine service, but they failed several appointments so his mother was advised to transfer care. His weight remains relatively stable, he continues to have troubles with sleep device use, and recently cancelled visits due to loss of health insurance.

## 6. Discussion

Each of these cases illustrates the complexity of providing medical care for youth with severe obesity. These cases demonstrate the many barriers patients and families face when trying to implement recommended care strategies. These youth required comprehensive care, including visits to multiple medical providers, psychosocial interventions, special schooling services, community resources, and CPS involvement.

The traditional treatment of obesity that focuses on increasing physical activity and making healthier dietary choices has not been successful for some of our patients, due to factors related to social determinants of health, such as family health issues, limited education and/or intellectual abilities and inadequate resources. As youth with severe obesity age, parents may lose control or “lose heart” while attempting to sustain healthy changes for everyone in the household. The physiology of excess adipose tissue drives persistence of and continued excessive weight gain [29,30]. Youth with severe obesity experience multiple medical comorbidities [15,16]. For example, increasing body habitus leads to physical limitations. Activities like self-care, climbing stairs, and participating in gym can become difficult. All of the subjects in our cases had difficulties with schooling, including learning delays, poor school compliance and the need for alternate school learning environments (online schooling, therapeutic school). Two of our subjects had sleep apnea and noncompliance with home sleep device use, which was likely contributing to poor school attendance, mental health issues, and weight gain [31,32]. Consistent psychiatric care had a positive impact on all 3 youth. Two cases reported loss of psychiatric services or therapy from issues related to insurance, clinic closure, or patient discharge due to failure to attend scheduled visits.

Keeping track of and attending the many appointments to several specialties was overwhelming for our youth and their families. Issues with transportation to visits were a recurring theme for 2 of the cases. Visits may require a parent to take the day off work (often without pay), making it especially difficult for families with inadequate financial resources. While medical visits are essential, we need to find ways to ease stress and the burden of multiple medical visits. We must find a balance between the necessity of patient monitoring and family/patient needs and limited resources. Many of the medical visits may not fully address the barriers to treatment that some families face. The resources in the community that may need to be involved include school, psychology services, food pantries, and housing alternatives. Extending our care beyond the medical setting requires careful coordination with community resources to ensure our patients have access.

To improve care of these youth, CPS was involved to advocate for the children to receive the services they needed. Similar to what others have found, the responsiveness of CPS varied [25]. When most responsive, CPS provided home visits, identified household needs, arranged transportation to medical visits, and placed a youth in a more structured environment (Case 2). There is another case report supporting the benefit of out of home placement for a child with severe obesity [33]. We worked with CPS to identify options for residential living outside of the family home for Cases 1 and 3, but were unable to identify any.

Caring for these youth and their families have taught us important lessons. Our failures to achieve optimal weight outcomes help reinforce our need to continue to improve our treatment strategies, including how to use meal replacements and medications to counteract the abnormal adipose physiology that is driving the maintenance of weight and the promotion of weight regain [34,35].

The review of these cases has heightened our awareness of the need to enhance motivation and work toward improved delivery of mental health services within our obesity treatment clinic. We also better understand the level of detailed information needed about the family and community to plan appropriate, individualized care strategies. We have learned that family and patient adherence to treatment strategies is not simply the result of being educated on healthy lifestyle habits, but is intricately intertwined with our patient’s psychosocial conditions which may need to be addressed before or in conjunction with medical treatments. These youth may have greatly benefited from bariatric surgery, but their mental health and medical issues precluded us from pursuing that option [36,37].

## 7. Conclusions

These case histories highlight the complexities of caring for youth with severe obesity. Care for these youth extends beyond targeting modifications of diet and physical activity behaviors. Low resource households and families, and social and mental health issues require multifaceted, coordinated care for these youth. Patients with serious obesity-related comorbidities require multiple medical visits with an array of providers. Oftentimes families do not have the resources to comply with provider expectations. The process of facilitating access to services outside of the medical setting, as was sought from CPS, can help identify and address household- and community-related barriers to successful treatment outcomes. However, our partnerships with CPS staff members were only sometimes successful. The review of these cases has helped us better understand the benefits and limitations of CPS involvement with our patients. It is our hope and our intent to use and share this information to guide our future approaches to meet the multifaceted needs of patients with severe obesity.
